# CYP2D6-inhibiting drugs and risk of fall injuries after newly initiated antidepressant and antipsychotic therapy in a Swedish, register-based case-crossover study

**DOI:** 10.1038/s41598-021-85022-x

**Published:** 2021-03-11

**Authors:** Marja-Liisa Dahl, Karin Leander, Max Vikström, Clara Frumerie, Sofia Nordenmalm, Jette Möller, Karin Söderberg-Löfdal

**Affiliations:** 1 Division of Clinical Pharmacology, Department of Laboratory Medicine, Karolinska Institutet, Karolinska University Hospital, Stockholm, Sweden; 2 Institute of Environmental Medicine, Karolinska Institutet, Stockholm, Sweden; 3 Department of Global Public Health, Karolinska Institutet, Stockholm, Sweden

**Keywords:** Risk factors, Trauma, Epidemiology, Adverse effects, Combination drug therapy, Epidemiology

## Abstract

Drug-drug interactions have been shown to affect the risk of fall injuries when opioids are used concomitantly with drugs inhibiting the cytochrome P450 2D6 (CYP2D6) enzyme in a previous pharmacoepidemiological study. The aim of this study was to determine whether CYP2D6-inhibiting drugs reinforce the risk of fall injuries when used concomitantly with antidepressants or antipsychotics. We identified all 252,704 adults with a first fall injury leading to hospitalisation from the National Patient Register in Sweden 2006–2013. Data on dispensed drugs was linked from the Swedish Prescribed Drug Register. We applied a case-crossover design to analyse newly dispensed (28 days preceding the fall injury, preceded by a 12-week washout period) antidepressants and antipsychotics, respectively, in relation to risk of a fall injury and according to concomitant use of CYP2D6-inhibiting drugs. Newly dispensed drugs were assessed correspondingly in a control period of equal length, 28 days prior to the 12-week washout period. Overall, the risk of fall injury was increased after newly initiated antidepressant and antipsychotic treatment. For antidepressants, concomitant CYP2D6 inhibitor use further elevated the risk estimates compared to non-use, most pronounced for the groups selective serotonin reuptake inhibitors (sertraline excluded) [OR = 1.47 (95% CI 1.19–1.80) vs. OR = 1.19 (95% CI 1.13–1.26)], and tricyclic antidepressants [OR = 1.71 (95% CI 1.17–2.51) vs. 1.27 (95% CI 1.11–1.47)] as well as for sertraline [OR = 1.61 (95% CI 1.05–2.38) vs. 1.12 (95% CI 1.00–1.26)]. For antipsychotics, the risk of fall injury was not altered by concomitant use of CYP2D6-inhibiting drugs. In conclusion, concomitant use of CYP2D6 inhibiting drugs tends to further increase the risk of fall injury in newly initiated antidepressant treatment, but not in antipsychotic treatment.

## Introduction

With ageing, the number of concomitant diseases and drugs increases, and hence also the risk of adverse drug effects such as falls. World-wide, approximately 30% of the population 65 years or older are estimated to fall yearly^1^. Fall injuries are more common among women and the eldest, and is often related to high mortality^2,3^. Further, persons hospitalised due to fall injury, compared to those not, had more hospitalisations the preceding year. Socioeconomic deprivation has also been associated with an increased incidence of fractures in the elderly, and more than 90% of the fractures follow low-energy falls^4^.

Fall-risk-increasing drugs (FRIDs) include drugs for cardiovascular diseases, benzodiazepines, antidepressants, antiepileptics, antipsychotics, antiparkinsonian drugs, opioids and urological spasmolytics^5^. Concomitant use of more than three medications, including psychotropics, cardiovascular drugs or analgesics increases the risk of fall^6^. In some studies, polypharmacy as such, irrespective of the use of FRIDs has also been shown to be an independent risk factor for falls^7^. However, in other studies only polypharmacy with at least one FRID was shown to increase the risk of falls^8,9^. Although polypharmacy may lead to harmful drug–drug interactions (DDIs), research on the clinical impact of DDIs at the population level is sparse. The risk of adverse effects due to DDIs increases with an increased number of concomitant medications^10–12^. In particular, metabolic drug interactions are common and potentially avoidable.

Antidepressants and antipsychotics are widely used psychotropic drugs, broadly defined as drugs acting directly on the central nervous system. It is estimated that 20% of the population 65 years and older use one or more such drugs, whereas in residential care the corresponding figure is as high as 80% in this age group; overall, approximately 37% use antidepressants and 46% antipsychotics^13^. For antidepressants and antipsychotics, increased risks of fall injuries have been found in the elderly^14,15^, with even a six-fold risk increase for fall injuries associated with the use of antidepressants^16^. However, the known association between psychotropic agents and falls is primarily based on observational data with only minor adjustments for confounders, medication doses, duration of the medication or polypharmacy. Several meta-analyses and studies have confirmed the association between falls and psychotropic drug use^6,15–22^. In previous studies, short periods of medication exposure were associated with elevated risks for fall injuries; 30 days for antidepressants^23^ and 3 months for psychotropic drugs including antidepressants with the fall risk found to be highest within 7 days of medication start among both men and women^24^. This is in accordance with the notion that adverse effects often are most pronounced when medications are newly initiated^10,25,26^.

In clinical practice today, 20–25% of all medications are estimated to be metabolised at least partly via the cytochrome P450 2D6 (CYP2D6). The elderly are more sensitive to drug actions due to altered physiology. Consequently, they may be more affected by elevated blood concentrations of drugs when elimination pathways are impaired due to inhibition of enzyme activity. Therefore, adverse effects such as sedation and nausea caused by antidepressants and antipsychotics can lead to fall injury^3,16^. However, there are only scarce population-based studies investigating whether metabolic DDIs affect adverse effects. For fall injuries, the only one is our previous case-crossover study where CYP2D6-inhibiting drugs were found to have an effect on the risk of falls when combined with an opioid metabolised via CYP2D6, compared to those with other metabolic pathways^27^. For many antidepressants and antipsychotics, CYP2D6 is the main pathway of metabolism, but the elimination is often complex and involves several CYPs and other drug metabolising enzymes as well^27–33^. Several drugs from different therapeutic groups are inhibitors of CYP2D6. In particular, strong inhibitors may give rise to increased plasma levels of other medications metabolised by CYP2D6 when used in combination^14,34^, which in turn may result in increased risks of adverse effects. Herein we hypothesise that concomitant use of CYP2D6-inhibiting drugs has an effect on risk of fall injury for psychotropic drugs metabolised via CYP2D6. In this study we aim to determine the risk of fall injury after newly initiated treatment with antidepressants or antipsychotics, and the impact of concomitant use of CYP2D6-inhibiting drugs on the risks in relation to gender, age and type of fall.

## Methods

### Data source

We conducted a case-crossover study using record-linkage between national registries covering the entire Swedish population. We retrieved data from the National Patient Register which covers all registered hospital discharges in the entire Swedish population since 1987; more than 99% of all hospital discharges^35^. The civic registration number assigned to all Swedish citizens makes it possible to link hospital discharge diagnoses and dates from the National Patient Register to drug dispensations. Individual-level information on prescribed dispensed drugs was retrieved from the Swedish Prescribed Drug Register (SPDR) which contains data on all dispensed prescribed drugs to the entire population of Sweden (10 million inhabitants), reported from the pharmacies since July 2005^36^. Antidepressants and antipsychotics are prescription drugs, not sold over the counter in Sweden. The SPDR includes data on drug substance, dispensation date and the Anatomical Therapeutic Chemical (ATC) classification code. Both registers have previously been used for a large number of pharmacoepidemiological studies with different designs^37^.

### Study design and setting

The study base is the entire adult Swedish population (20 years of age or older at index date) between 2006 and 2013. We employed a case-crossover design, where each fall injury patient also acts as his/her control^27,38^. The case-crossover design is analogous to a matched case–control design: however, instead of comparing cases with persons without the outcome, controls, each case contributes with his/her own control information from a period when no outcome occurred. To apply the case-crossover design on our case series of fall-injured patients, we utilised three types of information: time of injury; antidepressant/antipsychotic use during a defined period prior to the fall injury (case information); and antidepressant/antipsychotic use in a reference period (control information).An advantage is that between-subject differences in measured or unmeasured time-invariant confounders, such as chronic diseases and lifestyle are avoided. The risk of time-varying confounding will be smaller, the closer in time the control period is to the case period. The case-crossover design has been found useful for studying drug-use with acute onset of effect^11^.

### Study subjects

**Figure 1:**
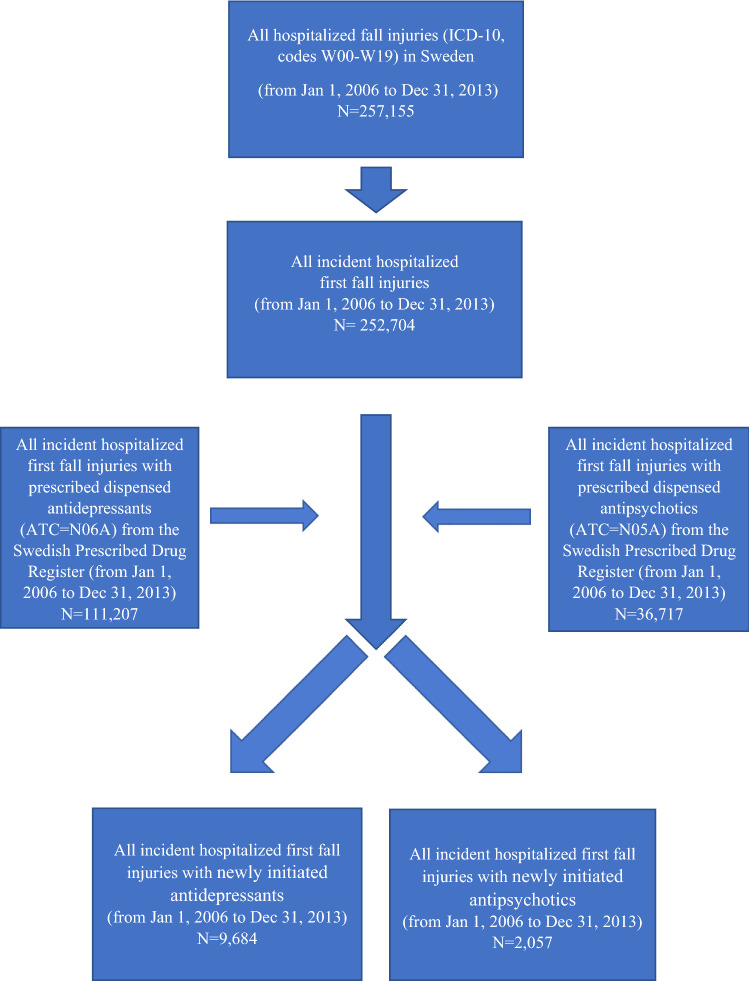
Flow chart of inclusion/exclusion criteria. *ICD-10* the International Classification of Diseases, 10th revision, *ATC* the anatomical therapeutic chemical classification.

*Study sample/case series.* The National Patient Register was used to obtain all hospitalised first fall injuries for an individual in the Swedish population during the study period from 1st of January 2006 to 31st of December 2013. Every patient is only included once in the dataset, i.e. the first time that they are hospitalised for a fall injury. Discharge diagnoses are coded according to *the International Classification of Diseases*, 10th revision (ICD-10, codes W00-W19). We identified 252,704 cases (Fig. 1). The index date was defined as the date of admission due to diagnosis of fall injury.

### Definition of exposure

Antidepressant and antipsychotic drugs used in Sweden during the study period were identified from the ATC groups N06A and N05A, respectively. For analyses, antidepressants were grouped as selective serotonin reuptake inhibitors (SSRI), tetracyclic antidepressants, tricyclic antidepressants, and serotonin-norepinephrine reuptake inhibitors (SNRI) metabolised at least to a certain degree by CYP2D6, and others, not metabolised by CYP2D6 (bupropion, reboxetine, moclobemide) (Table 1). Fluvoxamine was also considered to be included in the SSRI group, but it was not prescribed to any patients in our material. Sertraline was analysed separately from the other SSRIs as the role of CYP2D6 is uncertain, only shown in vitro. The SNRIs duloxetine, without active metabolites, and venlafaxine, with active metabolites, were also analysed individually. Similarly, antipsychotic drugs were grouped as those metabolised by CYP2D6 (chlorpromazine, perphenazine, haloperidol, zuclopenthixol), those with CYP2D6-formed active metabolites (risperidone, aripiprazole) and non-CYP2D6 metabolised antipsychotics (levomepromazine, prochlorperazine, melperone, ziprasidone, flupentixol, chlorprotixen, clozapine, quetiapine, paliperidone, olanzapine). In addition, the result from an extensive literature search carried out by the authors was used as a basis for subcategorization of the CYP2D6-metabolised drugs with respect to the impact of CYP2D6 metabolism on their clinical pharmacokinetics; ‘Major’ (antidepressants paroxetine, maprotiline, nortriptyline, amitriptyline, clomipramine, trimipramine and the antipsychotic perphenazine) and ‘Partial’ (antidepressants citalopram, escitalopram, fluoxetine, mianserin, duloxetine, mirtazapine and antipsychotics chlorpromazine, haloperidol, zuclopenthixol) (Tables 2, 3, 4, 5). Risperidone and aripiprazole were analysed together as a separate group, as the main metabolites formed by CYP2D6 (and CYP3A4) are pharmacologically active.

**Table 1:** Description of the fall injury cases by age group, sex, use of antidepressive drugs, antipsychotics and CYP2D6 inhibiting drugs in the 28-day period before index date, n = 252,704.

	Numbers	Percentage (%)
**Age group (years)**		
20–29	9139	3.6
30–39	8287	3.3
40–49	13,093	5.2
50–59	21,333	8.4
60–69	34,774	13.8
70–79	49,555	19.6
80–89	84,450	33.4
90 +	32,073	13.0
**Sex**		
Men	99,303	39.3
Women	153,401	60.7
**Antidepressants metabolised by CYP2D6**		
*SSRI*		
Paroxetine	135	0.1
Citalopram	2533	1.0
Escitalopram	248	0.1
Fluoxetine	111	0.0
*SSRI (CYP2D6 metabolism shown *in vitro*)*		
Sertraline	696	0.3
*Tetracyclics*		
Mianserin	71	0.0
Maprotiline	6	0.0
Mirtazapine^a^	808	0.3
*Tricyclics*		
Nortriptyline^a^	11	0.0
Amitriptyline^a^	417	0.2
Clomipramine^a^	83	0.0
Trimipramine^a^	7	0.0
*SNRI*		
Duloxetine	95	0.0
Venlafaxine^a^	206	0.1
**Non CYP2D6 metabolised antidepressants**		
Bupropion	39	0.0
Reboxetine	12	0.0
Moclobemide	1	0.0
**Antipsychotics metabolised by CYP2D6**		
*with active metabolites*		
Risperidone^a^	463	0.2
Aripiprazole^a^	18	0.0
*Major CYP2D6 metabolism*		
Perphenazine	38	0.0
*Partial CYP2D6 metabolism*		
Chlorpromazine	2	0.0
Haloperidol	293	0.1
Zuclopenthixol	42	0.0
**Non CYP2D6 metabolised antipsychotics**		
Levomepromazine^a^	82	0.0
Prochlorperazine	31	0.0
Melperone	27	0.0
Ziprasidone	4	0.0
Flupentixol	46	0.0
Chlorprotixen	8	0.0
Clozapine	20	0.0
Quetiapine	64	0.0
Paliperidone	1	0.0
Olanzapine	121	0.0
**CYP2D6 inhibitors**	32,714	12.2
Strong	1632	0.6
Moderate	5059	2.0
Weak	12	0.0
Unspecified	26,857	10.6

Substances marked with ^a^have active metabolites.

The Flockhart cytochrome P450 drug interactions table^33^ was used to identify and classify strong (bupropion, cinacalcet, fluoxetine, paroxetine, quinidine), moderate (duloxetine, sertraline, terbinafine), weak (amiodarone, cimetidine) or unspecified (celecoxib, chlorpromazine, citalopram, clemastine, clomipramine, diphenhydramine, doxorubicin, escitalopram, haloperidol, hydroxyzine, levomepromazine, methadone, metoclopramide, moclobemide, perphenazine, ranitidine, ritonavir, ticlopidine) CYP2D6 inhibitors.

**Figure 2:**
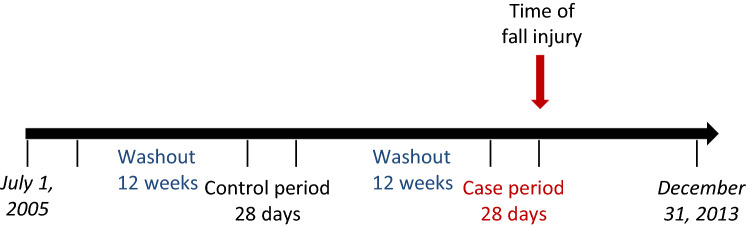
Graphical presentation of the design, definitions of case, control and washout periods.

For a patient to be classified as newly exposed, the drug prescription had to be dispensed within the 28 days preceding the injury (so called case-period, see Fig. 2). In addition, to ascertain that the therapy was newly initiated we used a 12-week washout period prior to the start of the 28 day period, with no dispensations of another antidepressant when the effect of antidepressants was analysed, or another antipsychotic medication when exposure was antipsychotics. Similarly, exposure was assessed for the control-period (112–140 days before the fall injury). The length of the washout periods was chosen to ascertain that antidepressant/antipsychotic exposure is newly initiated. In Sweden, the maximum amount of drug prescribed for each dispensation is 3 months.

For descriptive purposes, we retrieved national data on sociodemographic factors from Statistics Sweden’s data base on the aggregated level for all persons 20 years or older. Statistics Sweden is responsible for official statistics for sociodemographic factors and for other government statistics and coordinate the system for the official statistics in Sweden. This data was used to rank all municipal areas in several sociodemographic aspects to describe the usage of antidepressants and antipsychotics in the study population from which the fall injury cases are retrieved, in order to facilitate comparisons and generalisability to other populations. The dispensation of antidepressants and antipsychotics in the 20 municipalities with the lowest and highest incidence of fall injuries, and lowest and highest level of education (percentage of the population with at least three years of post-secondary education) and percentage foreign born were calculated for the years 2006 and 2013 (Supplementary 2).

### Possible confounders

We obtained data on the use of other drugs from the SPDR to adjust for confounding from newly initiated treatment with other fall-inducing drugs: antiepileptics (N03A), beta-blockers (C07), opioids (N02A), anxiolytics (N05B), and hypnotics and sedatives (N05C) using the same for exposure in the case- and control-periods as for antidepressant/antipsychotic exposure (see 2.6). In addition, we adjusted for antipsychotics (N05A) in our analyses of antidepressants, and for antidepressants (N06A) in our analyses of antipsychotics.

### Statistical analysis

Analyses were performed with standard methods for matched case–control studies^38^. Each case was considered one stratum and conditional logistic regression was used to estimate odds ratios (OR) as measures of relative risks^11,38^. The results are presented as OR with 95% confidence interval (95% CI) for the case-period with the length of 28 days. Sensitivity analyses were performed for a case-period with the length of 14 days to examine whether a shorter period of use would alter the results. Even shorter time periods were not adequate due to small numbers resulting in lack of precision. The same individuals were observed and compared within themselves, i.e. the case-period with a corresponding period without the event (control period) (see Fig. 2). The same length of washout period, 12 weeks, was used before the control-period. We stratified for use of any CYP2D6–inhibiting drug (strong, moderate, weak or unspecified), and for strong or moderate CYP2D6-inhibiting drugs^33^. Stratified analyses were done for sex and age, with the age groups categorized as: 20–69, 70-, 80- and according to type of fall: from low level (ICD-10 codes: W00-09, W18), from high level (W10-W17) and unspecified (W19). The age categories 70- and 80- are not mutually exclusive as the idea was to separately study the elderly while maintaining sufficient number of participants for analysis. In addition, we restricted analyses to fall injuries with femur neck (S72.0) or pertrochanteric (S72.1) fractures, as sensitivity analyses to address reversed causality, as these diagnoses require immediate hospitalization and surgical procedures. All statistical analyses were performed using the statistical software SAS (version 9.4, SAS institute, Cary, NC, USA).

### Ethical approval

The project has approval from the Regional Ethical Review Board in Stockholm (2014/856-31, 2015/837-32).

## Results

In Table 1, descriptive data of fall injured (n = 252,704) is shown by age group, sex, newly initiated antidepressants and antipsychotics as well as CYP2D6 inhibiting drugs in the 28 days preceding the fall injury. The majority of the fall injuries occurred among elderly (70 years and older) and was most frequent in the age group 80–89 years (33.4%). The mean age at time of fall injury was 67.2 years for men and 76.1 years for women. Overall, 5,321 cases (2.1%) used any antidepressant and 1218 (0.48%) any antipsychotic drug. Women constituted 60.7% of the fall injured, 69.6% of newly initiated antidepressant use and 62.8% of newly initiated antipsychotic use. The use of antidepressants and antipsychotics stratified for men and women in 10-year age groups is described in Supplementary 1.

Sociodemographic factors [educational level and country of birth (Sweden or foreign)] related to antidepressant and antipsychotic dispensation on aggregated population level are presented in Supplementary 2. For the sociodemographic factors studied, dispensations of antidepressants and antipsychotics decreased from 2006 to 2013. For antidepressants, the decrease was proportional and in the same direction for municipalities with the highest and lowest incidence of fall injuries. In 2006, the 20 municipalities with the highest incidence had a three times higher number of dispensed antipsychotics per 10 000 inhabitants compared to the 20 with the lowest fall incidence. In 2013, the difference had decreased but was still two-fold. High education municipalities showed decreased antidepressant dispensation in 2013 compared to 2006 while the pattern was mixed in low education municipalities. Antidepressants were less dispensed in municipalities with a higher proportion of foreign-born inhabitants in both 2006 and 2013. For antipsychotics, the patterns were quite similar comparing educational level or proportion foreign born inhabitants. The trends were consistent over the years, i.e. the sociodemographic groups with the highest dispensations, in general, had the highest numbers in both 2006 and 2013.

The results for fall injury after newly initiated drugs stratified by concomitant use of CYP2D6-inhibiting drugs are shown in Table 2 for antidepressants, and in Table 5 for antipsychotics.

**Table 2:** Risk of fall injury after newly initiated antidepressive treatment in the case period (1–28 days prior to index date) and the control period (112–140 days prior to index date) stratified by concomitant use of CYP2D6-inhibiting drugs 28 days prior to index date.

Drug names	CYP2D6-inhibiting drugs								
	No			Yes (all)			Yes (strong and moderate)		
	N_case_	N_control_	R (95% CI)	N_case_	N_control_	R (95% CI)	N_case_	N_control_	R (95% CI)
**Antidepressants metabolized by CYP2D6**									
SSRI									
Paroxetine, Citalopram, Escitalopram, Fluoxetine	2799	2349	1.19 (1.13–1.26)	227	155	1.47 (1.19–1.80)	27	24	1.13 (0.65–1.95)
Sertraline	629	561	1.12 (1.0–1.26)	66	41	1.61 (1.05–2.38)	66	22	3.00 (1.85–4-86)
**Tetracyclics**									
Mianserin, Maprotiline, Mirtazapine	714	580	1.23 (1.10–1.37)	171	120	1.43 (1.13–1.80)	44	25	1.76 (1.08–2.85)
**Tricyclics**									
Nortriptyline, Amitriptyline, Clomipramine, Trimipramine	446	350	1.27 (1.11–1.47)	72	42	1.71 (1.17–2.51)	11	11	1.00 (0.43–2.31)
**SNRI**									
Duloxetine	91	60	1.52 (1.10–2.10)	4	7	0.57 (0.17–1.95)	4	3	1.33 (0.30–5.96)
Venlafaxine	188	186	1.01 (0.83–1.24)	18	22	0.82 (0.44–1.53)	2	6	0.33 (0.07–1.65)
**No CYP2D6 metabolism**									
Bupropion, Reboxetine, Moclobemide	41	32	1.28 (0.81–2.03)	11	9	1.22 (0.51–2.95)	7	5	1.40 (0.44–4.41)
**Major CYP2D6 metabolism**									
Paroxetine, Maprotiline, Nortriptyline, Amitriptyline, Clomipramine,Trimipramine	581	466	1.25 (1.10–1.41)	78	48	1.63 (1.13–2.33)	17	12	1.42 (0.68–2.97)
**Partial CYP2D6 metabolism**									
Citalopram, Escitalopram, Fluoxetine, Mianserin, Duloxetine, Mirtazapine	3468	2837	1.22 (1.15–1.28)	330	235	1.40 (1.19–1.66)	64	43	1.49 (1.01–2.19)
**Combined groups CYP2D6 metabolised (major + partial)**	4044	3296	1.23 (1.16–1.27)	377	266	1.42 (1.21–1.66)	70	49	1.43 (0.99–2.06)

*N*_*case*_ refers to number of subjects with antidepressants dispensed in the case period and not in the control period; *N*_*control*_ refers to number of cases with newly initiated antidepressants dispensed in the control period and not in the case period; *OR,* odds ratio; *CI,* confidence interval; SSRI (selective serotonin reuptake inhibitors); SNRI (selective serotonin-norepinephrine reuptake inhibitors); Venlafaxine is not included in a CYP2D6 metabolism group as it has active metabolites.

### Antidepressants

Overall, we found increased risks of fall injuries after newly initiated therapy with antidepressant drugs, both with and without concomitant use of CYP2D6-inhibiting drugs (Table 2). For antidepressant groups, where the number of cases produced relatively robust results (at least 20 observed cases), all risk estimates were higher with concomitant use of CYP2D6-inhibiting drugs than without. The most elevated risks with concomitant use of any CYP2D6-inhibiting drug were found for the SSRI group (sertraline excluded), sertraline, tetracyclics and tricyclics. For antidepressants with partial or major metabolism by CYP2D6, the ORs were further increased when CYP2D6-inhibiting drugs were also prescribed.

If we assume that the odds ratios we have calculated are reasonable estimates of the relative risks, our results mean that newly deployed SSRI drug (sertraline excluded) with concomitant use of CYP2D6-inhibiting drug, accounts for 200 additional cases per 100,000 individuals in our population and year, whereas the same drug exposure although without concomitant use of CYP2D6-inhibitor accounts for 85 additional cases. The corresponding numbers for sertraline and tricyclics are 260 additional cases versus 52, and 310 additional cases versus 120. The yearly total number of cases in the population is around 32,000 per 100,000 individuals.

With restriction to strong and moderate CYP2D6-inhibiting drugs and at least 20 observed cases, compared to all CYP2D6-inhibiting drugs the risk estimate was lower for SSRI while the risk estimates were further elevated for sertraline and tetracyclics (Table 2). The risk estimates were also further increased for antidepressants with partial CYP2D6 metabolism as well as when those with major or partial CYP2D6 metabolism were combined.

In women, the increased risks for sertraline with concomitant use of CYP2D6-inhibiting drugs were pronounced and further emphasized with restriction to strong and moderate inhibitors (Table 3). In contrast, elevated risks with concomitant use of any CYP2D6 -inhibiting drugs were pronounced in men for antidepressants with major, partial (and the combined major/partial) CYP2D6 metabolism with the highest risk in the subgroup with major CYP2D6 metabolism (Table 4).

**Table 3:** Risk of fall injury after newly initiated antidepressive treatment in women in the case period (1–28 days prior to index date) and the control period (112–140 days prior to index date) stratified by concomitant use of CYP2D6-inhibiting drugs 28 days prior to index date.

Drug names	CYP2D6-inhibiting drugs								
	No			Yes (all)			Yes (strong and moderate)		
	N_case_	N_control_	OR (95% CI)	N_case_	N_control_	OR (95% CI)	N case	N control	OR (95% CI
**Antidepressants metabolized by CYP2D6**									
SSRI									
Paroxetine, Citalopram, Escitalopram, Fluoxetine	1993	1625	1.23 (1.15–1.31)	140	105	1.33 (1.04–1.72)	15	15	1.00 (0.49–2.05)
Sertraline	435	396	1.10 (0.96–1.26)	49	20	2.45 (1.46–4.12)	49	9	5.44 (2.68–11.08)
**Tetracyclics**									
Mianserin, Maprotiline, Mirtazapine	452	383	1.18 (1.03–1.35)	116	84	1.38 (1.04–1.83)	34	16	2.13 (1.17–3.85)
**Tricyclics**									
Nortriptyline, Amitriptyline, Clomipramine, Trimipramine	333	278	1.20 (1.02–1.41)	49	34	1.44 (0.93–2.32)	6	9	0.67 (0.24–1.87)
**SNRI**									
Duloxetine	62	41	1.51 (1.02–2.24)	4	6	0.67 (0.19–2.36)	4	3	1.33 (0.30–5.96)
Venlafaxine	125	118	1.06 (0.82–1.36)	12	13	0.92 (0.42–2.02)	1	4	0.25 (0.03–2.24)
**No CYP2D6 metabolism**									
Bupropion, Reboxetine, Moclobemide	21	21	1.00 (0.55–1.83)	6	6	1.00 (0.32–3.10)	3	5	0.60 (0.14–2.51)
**Major CYP2D6 metabolism**									
Paroxetine, Maprotiline, Nortriptyline, Amitriptyline, Clomipramine, Trimipramine	435	367	1.19 (1.03–1.36)	52	38	1.37 (0.90–2.08)	9	9	1.00 (0.40–2.50)
**Partial CYP2D6 metabolism**									
Citalopram, Escitalopram, Fluoxetine, Mianserin, Duloxetine, Mirtazapine	2401	1937	1.24 (1.17–1.32)	212	159	1.33 (1.09–1.64)	45	29	1.55 (0.97–2.48)
**Combined groups CYP2D6 metabolised (major + partial)**	2836	2290	1.24 (1.17–1.31)	243	183	1.33 (1.10–1.61)	48	33	1.46 (0.93–2.27)

*N*_*case*_ refers to number of subjects with antidepressants dispensed in the case period and not in the control period; *Ncontrol* refers *to* number of cases with antidepressants dispensed in the control period and not in the case period; *OR,* odds ratio; *CI,* confidence interval; SSRI (selective serotonin reuptake inhibitors); SNRI (selective serotonin-norepinephrine reuptake inhibitors); Venlafaxine is not included in a CYP2D6 metabolism group as it has active metabolites.

**Table 4:** Risk of fall injury after newly initiated antidepressive treatment in men in the case period (1–28 days prior to index date) and the control period (112–140 days prior to index date) stratified by concomitant use of CYP2D6-inhibiting drugs 28 days prior to index date.

Drug names	CYP2D6-inhibiting drugs								
	No			Yes (all)			Yes (strong and moderate)		
	N_case_	N_control_	OR (95% CI)	N_case_	N_control_	OR (95% CI)	N case	N control	OR (95% CI
**Antidepressants metabolized by CYP2D6**									
SSRI									
Paroxetine, Citalopram, Escitalopram,Fluoxetine	806	724	1.11 (1.01–1.23)	87	50	1.74 (1.23–2.46)	12	9	1.33 (0.56–3.16)
Sertraline	192	165	1.16 (0.95–1.43)	17	21	0.81 (0.43–1.53)	17	13	1.31 (0.64–2.69)
**Tetracyclics**									
Mianserin, Maprotiline, Mirtazapine	262	197	1.33 (1.11–1.60)	55	36	1.53 (1.99–2.33)	10	9	1.11 (0.45–2.73)
**Tricyclics**									
Nortriptyline, Amitriptyline, Clomipramine, Trimipramine	113	72	1.57 (1.17–2.11)	23	8	2.88 (1.29–6.43)	5	2	2.50 (0.49–12.89)
**SNRI**									
Duloxetine	29	19	1.53 (0.86–2.72)	0	1	N/A	0	0	N/A
Venlafaxine	63	68	0.93 (0.66–1.31)	6	9	0.67 (0.24–1.87)	1	2	0.50 (0.05–5.51)
**No CYP2D6 metabolism**									
Bupropion, Reboxetine, Moclobemide	20	11	1.82 (0.87–3.80)	5	3	1.67 (0.40–6.97)	4	0	N/A
**Major CYP2D6 metabolism**									
Paroxetine, Maprotiline, Nortriptyline, Amitriptyline, Clomipramine, Trimipramine	146	100	1.46 (1.13–1.88)	26	9	2.89 (1.35–6.17)	8	3	2.67 (0.71–10.05)
**Partial CYP2D6 metabolism**									
Citalopram, Escitalopram, Fluoxetine, Mianserin, Duloxetine, Mirtazapine	1067	900	1.19 (1.09–1.30)	118	76	1.55 (1.16–2.07)	19	14	1.36 (0.68–2.71)
**Combined groups CYP2D6 metabolised (major + partial)**	1208	1000	1.21 (1.11–1.31)	134	83	1.61 (1.23–2.12)	22	16	1.38 (0.72–2.62)

The antidepressants are classified according to CYP2D6 metabolism *N*_*case*_ refers to number of subjects with antidepressants dispensed in the case period and not in the control period; *N*_*control*_ refers to number of cases with antidepressants dispensed in the control period and not in the case period; *OR,* odds ratio; *CI,* confidence interval; SSRI (selective serotonin reuptake inhibitors); SNRI (selective serotonin-norepinephrine reuptake inhibitors); Venlafaxine is not included in a CYP2D6 metabolism group as it has active metabolites.

Results from age-stratified analyses revealed further increased risks compared to the main results given in Table 2 with, but not without, concomitant use of CYP2D6-inhibiting drugs among the young/middle-aged (20–69 years) for SSRIs (sertraline excluded) [OR = 1.91 (95% CI 1.32–2.76) versus OR = 1.08 (95% CI 0.96–1.21)], and for tricyclic antidepressants [OR = 2.46 (95% CI 1.22–4.95) versus OR = 1.15 (95% CI 0.89–1.50)]. Similarly, in this age group, the ORs were higher with concomitant use of CYP2D6-inhibiting drugs compared to non-use for antidepressants with major [OR = 2.21 (95% CI 1.18–4.16) versus OR = 1.26 (95% CI 1.01–1.56)] and partial CYP2D6-metabolism [OR = 1.61 (95% CI 1.19–2.16) versus OR = 1.08 (95% CI 0.97–1.20)].

Among persons aged 70 years or older, the risk with concomitant CYP2D6-inhibitor use for SSRIs was somewhat elevated [OR = 1.30 (95% CI 1.01–1.66)], still elevated for sertraline [OR = 1.75 (95% CI 1.06–2.89)] but not significantly elevated for tricyclics [OR = 1.45 (95% CI 0.92–2.29)]. When we restricted to the age group of 80 years or older, concomitant CYP2D6-inhibitor use did not increase the risks significantly for SSRIs [OR = 1.23 (95% CI 0.92–1.65)], sertraline [OR = 1.11 (95% CI 0.59–2.06)] or tricyclics [OR = 1.22 (95% CI 0.66–2.28)]. For the antidepressants with major or partial CYP2D6-metabolism increased risks were found in the young/middle-aged group, while in both the oldest age groups (70- and 80-) risks were attenuated (data not shown).

### Antipsychotics

For antipsychotics, risks of fall injury were increased in all groups and similar with and without concomitant use of CYP2D6-inhibiting drugs (Table 5). The most elevated risk was seen for perphenazine with concomitant use of CYP2D6-inhibiting drugs, however, based on very few subjects. The results were similar for men and women (data not shown).

**Table 5:** Risk of fall injury after newly initiated antipsychotic treatment in the case period (1–28 days prior to index date) and the control period (112–140 days prior to index date) stratified by use of CYP2D6-inhibiting drugs 28 days prior to index date.

Drug names	CYP2D6-inhibiting drugs								
	No			Yes (all)			Yes (strong and moderate)		
	N_case_	N_control_	OR (95% CI)	N_case_	N_control_	OR (95% CI)	N_case_	N_control_	OR (95% CI)
**CYP2D6 metabolism with active metabolites**									
Risperidone, Aripiprazole	315	203	1.55 (1.30–1.85)	166	96	1.73 (1.35–2.22)	29	13	2.21 (1.16–4.29)
**Major CYP2D6 metabolism**									
Perphenazine	27	21	1.29 (0.73–2.27)	11	3	3.67 (1.02–13.14)	1	1	1.00 (0.06–16.00)
**Partial CYP2D6 metabolism**									
Chlorpromazine, Haloperidol, Zuclopenthixol	242	162	1.49 (1.36–1.95)	94	58	1.62 (1.17–2.25)	17	19	0.90 (0.47–1.72)
**Combined groups CYP2D6 metabolism (major + partial)**									
Perphenazine, Chlorpromazine, Haloperidol, Zuclopenthixol	269	183	1.47 (1.22–1.77)	105	61	1.72 (1.26–2.36)	18	20	0.90 (0.48–1.70)
**No CYP2D6 metabolism**									
Levomepromazine, Prochlorperazine, Melperone, Ziprasidone, Flupentixol, Chlorprotixen, Clozapine, Quetiapine, Paliperidone, Olanzapine	265	234	1.32 (0.95–1.35)	129	95	1.35 (1.04–1.77)	37	28	1.32 (0.81–2.16)

The antipsychotics are classified according to CYP2D6 metabolism. *N*_*case*_*,* refers to number of subjects with antipsychotic drugs dispensed in the case period and not in the control period; *N*_*control*_, number of cases with antipsychotic drugs dispensed in the control period and not in the case period; *OR,* odds ratio; *CI,* confidence interval. Risperidone is not included in the group with combined major metabolism as it has an active metabolite.

Analyses of separate age groups showed that in the young/middle-aged age group (20–69 years), the risk estimate for the groups of antipsychotics with active metabolites (risperidone and aripiprazole) was 3.43 (95% CI 1.48–7.96) with CYP2D6-inhibiting drugs and 0.75 (95% CI 0.41–1.38) without. In persons aged 70 years or older, the corresponding risk estimates for those metabolised by CYP2D6 (chlorpromazine, perphenazine, haloperidol, zuclopenthixol), were 2.05 (95% CI 1.42–2.96) with use of CYP2D6-inhibiting drugs and 1.71 (95% CI 1.38–2.71) without. In the same age group, the risks for non-CYP2D6 metabolised antipsychotics were 1.72 (95% CI 1.20–2.47) with and 1.14 (95% CI 0.92–1.42) without use of CYP2D6-inhibiting drugs. Otherwise, risks were not altered by age.

### Sensitivity analyses

In sensitivity analyses with restriction to 14 days of exposure, and all CYP2D6-inhibiting drugs, risk estimates were further increased for sertraline [OR = 2.11 (95% CI 1.21–3.70)], tricyclics [OR = 2.06 (95% CI 1.15–3.68)] and antidepressants with major CYP2D6 metabolism [OR = 1.83 (95% CI 1.10–3.04)] compared to 28 days of use while attenuated risks were found for antipsychotics. For antidepressants, separate analyses of different types of falls did not affect the results except for unspecified falls for which a tendency of even more pronounced risks with concomitant use of CYP2D6-inhibiting drugs was found for tricyclics [OR = 3.33 (95% CI 1.34–8.30)] compared to non-use [OR = 1.26 (95% CI 0.95–1.67)], and for those with major CYP2D6 metabolism [OR = 3.29 (95% CI 1.41–7.66)] versus [OR = 1.23 (95% CI 0.97–1.56)]. For antipsychotics, separate analyses of different types of falls did not alter the risks. For both antidepressants and antipsychotics, restriction to femur neck and pertrochanteric fractures or control from confounding did not alter the results, with the exception of antidepressants that decreased the risk for the group risperidone and aripiprazole with concomitant use of CYP2D6-inhibiting drugs [OR 1.11 (95% CI 0.82–1.49)].

## Discussion

We observed associations between newly initiated antidepressant or antipsychotic treatment and the risk of first-time fall injury both with and without concomitant use of CYP2D6-inhibiting drugs. For antidepressants, concomitant use of CYP2D6-inhibiting drugs tended to further increase the risk of fall injuries. To the best of our knowledge, this large (> 250,000 cases) population-based pharmacoepidemiological study is the first investigating clinical consequences of DDIs between newly initiated Central Nervous System (CNS) active drugs and CYP2D6-inhibiting drugs. CYP2D6-inhibiting drugs tended to further elevate the risk of fall injury in newly initiated antidepressant treatment. As most antidepressants are metabolised at least partly via CYP2D6, this would be in accordance with increased concentrations of the active drug in blood when its metabolism is inhibited, equivalent to intake of higher doses. The most pronounced risk estimates with concomitant use of CYP2D6-inhibiting drugs were shown for the groups of SSRIs, tetracyclics and tricyclics and in particular for sertraline when used concomitantly with strong or moderate inhibitors of CYP2D6. Sertraline was analysed separately from the other SSRIs as CYP2D6 metabolism has only been shown in vitro^39^ but not in vivo^40^. In women, but not men, the risks of fall injury for sertraline were increased with a suggested dose–response association for CYP2D6 inhibition as the risk estimate was increased with concomitant use of CYP2D6-inhibiting drugs and further increased with restriction to concomitant use with strong or moderate CYP2D6-inhibiting drugs. Our results are thus surprising and could be interpreted as indicative of the role of CYP2D6 also in the clinical pharmacokinetics of sertraline. The risk estimates were further increased for sertraline, tricyclics and for all antidepressants with major CYP2D6 metabolism when exposure was restricted to 14 days. This strengthens the observation that newly initiated therapy increases the risk of fall injury as adverse effects often are more pronounced when medication starts^10,25,26^. Psychotropic drugs have been seen to increase the risk of fall injury fivefold when started one week prior to index date compared to 1.4-fold when started 8–85 days before index date^24^.

For antipsychotics, CYP2D6-inhibiting drugs did not clearly modify the risk of fall injury overall except for a three-fold risk increase for the group of risperidone and aripiprazole in the young/middle-aged age group. The results could be interpreted as that antipsychotics per se are more important than CYP2D6 inhibition in affecting the risk of fall injury in newly initiated treatment.

The increased risk for fall injury for sertraline after concomitant use of CYP2D6-inhibiting drugs was only present among women. The number of cases was higher for women than for men which could influence the results. Women have been shown to reach somewhat higher sertraline concentrations in blood per dose^41^ which as such might increase the risk for adverse effects.

In sub-analyses of antidepressants stratified in age groups, in the older age groups, the elevated risks in the main results for SSRI, tricyclics and combined major and/or partial CYP2D6 metabolised antidepressants were attenuated or no longer significant (the confidence interval included one). The different effect in different age groups could be interpreted as that other factors such as decreased physiological compensatory mechanisms, other diseases and polypharmacy are more important for fall injury risk in the elderly. It is also possible that in the elderly, lower doses of antidepressants are used, as such decreasing the risk of falls as adverse effects of the drugs.

For antipsychotics, separate analyses of the young/middle-aged group (20–69 years) revealed an elevated risk of fall injury for the risperidone/aripiprazole group with use of concomitant CYP2D6-inhibiting drugs. Both these drugs are metabolised by CYP2D6, but also have pharmacologically active metabolites. In persons at least 70 years of age, a tendency for an increased association of fall injury for CYP2D6-metabolised antipsychotics with concomitant CYP2D6-inhibiting drugs was found. However, such a tendency though less pronounced was also seen for non-CYP2D6 metabolised antipsychotics which makes it difficult to assess the relevance of these findings. Otherwise, risk estimates were further elevated for antipsychotics metabolised by CYP2D6 both with and without use of CYP2D6-inhibiting drugs.

We investigated if type of fall differed in risks as fall from a low level has been suggested as a proxy for (an indicator representing) the most fragile elderly where medication overdose and fear of falling are important risk factors^42^. Additionally, patients who suffer falls from a low level have previously been shown to be at higher risk of 12-month mortality independently of age and co-morbidities^43^. However, in our study risks did not differ substantially between different age groups, which suggests that the association between the investigated drugs and fall injuries among the most fragile elderly is the same as in other subpopulations. It could be speculated this is due to good adherence to national guidelines to avoid certain medications to the elderly and adjusted dosage regimens resulting in less adverse effects^3^. However, a tendency of pronounced associations for unspecified falls with concomitant use of CYP2D6-inhibiting drugs was found for tricyclics and for antidepressants with major CYP2D6 metabolism. For antipsychotics, the effect estimates were the same regardless of type of fall. The results indicate that CYP2D6-inhibiting drugs affect fall risk to a greater extent when combined with newly initiated antidepressants than with antipsychotics. However, the increased risk with use of antipsychotics with CYP2D6-formed active metabolites was explained by confounding from newly initiated antidepressants.

Our results for tetracyclics and tricyclics are consistent with those based on nursing-home residents where a significant increase in acute fall-risk was seen with non-SSRI antidepressants [OR = 4.7 (95% CI 1.3–16.2)], but not for SSRIs as risk was not affected among the nursing-home residents^18^. Further, in meta-analyses, psychotropic drugs as a group have been shown to increase the risk of falls^6,15–22^. There is evidence of a reduced rate of falls when gradually withdrawing psychotropic drugs^17^. However, the discontinuation of antipsychotic drugs is not always an alternative, confirming the importance of elucidating potential adverse clinical effects due to DDIs, which was the purpose of our study.

It is plausible that our findings can be generalized to falls that are less severe because the hypothesized contribution of DDIs to the risk of falls would not be expected to differ depending on severity of falls.

There is an increasing awareness of the clinical importance to consider adverse effects resulting from DDIs^44^. Several studies show adverse effects when combining perphenazine and CYP2D6 inhibiting drugs, supporting our result where we see an increased fall-risk with such combinations^33,45,46^. However, studying potential pharmacokinetic DDIs on an epidemiological level is this far a quite new approach^27^.

Our study has several strengths. First, misclassification of outcome is unlikely as the fall injuries are diagnosed by a physician in a hospital setting. We utilized nationwide population-based registries with high coverage and high validity: more than 99% of all hospital discharge diagnoses are covered and the validity of fall injury diagnoses is 94% (35). Second, our register-based assessment of drug exposure is another strength: the coverage of the SPDR is complete for all dispensed prescribed drugs in Sweden^36^. Further, antidepressants and antipsychotics are not sold over the counter in Sweden. Third, by using the case-crossover design, we were able to take into account both measurable and unmeasurable time-invarying confounders^47^. Despite the advantage of the chosen study design in terms of confounding, we cannot rule out confounding from other potentially fall-inducing drugs; some of the CYP2D6-inhibiting drugs could as such increase the risk of falls and the interaction thus be pharmacodynamic rather than pharmacokinetic. However, for antipsychotics not metabolised by CYP2D6, the risk estimates were similar with and without CYP2D6 inhibitor use, indicative of lack of pharmacodynamic interaction. For antidepressants, the numbers of cases with non-CYP2D6 metabolised drugs were too small to allow any conclusions in this respect.

A limitation with the present study is potential misclassification of exposure due to poor compliance to prescribed and dispensed drugs. Still, the data collected is based on pharmacy dispensations, i.e., the patients have collected the prescribed drugs at the pharmacy, which is one step closer to use than prescription data. In addition, patients are most compliant when medication just started^48,49^. Also, we can not exclude some bias if seasonality in fractures and depressions are correlated. However, fractures are increased in winter while depressions tend to increase in autumn which would rather increase exposure in the control period and thus risks would be underestimated. Another study limitation is related to that the SPDR does not include information on drugs used in hospitals. However, most antidepressants and antipsychotics are prescribed in the outpatient setting. Reassuring is also that results from our analyses restricted to fall diagnoses that require immediate hospitalization and surgery show similar results as our main analyses and hence rule out the risk of reversed causation related to other drugs.

## Conclusions

We found that newly initiated antidepressants increased the risk of fall injuries, and concomitant use of CYP2D6-inhibiting drugs tended to further increase this risk; the most pronounced risks were found for SSRIs including sertraline, tetracyclics and tricyclics. In contrast, CYP2D6-inhibiting drugs did not alter the increased risk of fall injury observed for newly initiated antipsychotic therapy. To facilitate rational drug use, it is crucial to consider polypharmacy and possible DDIs that could increase the risk of adverse effects.

## Supplementary Information

Supplementary Tables.

Supplementary Figures.

## Data Availability

The data in this study are retrieved from the Swedish National Patient Registry and Swedish Prescribed Drug Registry, the ethical permission restricts further distribution of data.
