# Supplementary material for: CYP2D6-inhibiting drugs and risk of fall injuries after newly initiated antidepressant and antipsychotic therapy in a Swedish, register-based case-crossover study
Source: Sci Rep. 2021 Mar 11;11:5796. doi: 10.1038/s41598-021-85022-x (PMC7970948; doi:10.1038/s41598-021-85022-x)
Supplement: Supplementary file 1 — Supplementary Tables. [file 41598_2021_85022_MOESM1_ESM.pdf]

Supplementary 1 (A). Use of antidepressant substances stratified for men and women in 10-year age groups.

ATC code	Antidepressants			20-29 years		30-39 years		40-49 years		50-59 years		60-69 years		70-79 years		80-89 years		90- years	
		Men (total)	Women (total)	Men	Women	Men	Women	Men	Women	Men	Women	Men	Women	Men	Women	Men	Women	Men	Women
	SSRI																		
N06AB03	Fluxoxetine	30	81	4	5	4	6	5	12	5	6	5	14	3	17	4	15	0	6
N06AB04	Citalopram	753	1780	6	5	15	14	19	36	36	73	87	168	190	387	324	792	76	305
N06AB05	Paroxetine	35	100	1	2	5	3	2	22	9	15	5	25	6	28	3	5	4	0
N06AB10	Escitalopram	76	172	1	4	3	8	4	8	10	16	12	28	14	38	27	63	5	7
	**Total**	**893**	**2133**																
N06AB06	Sertraline	209	486	15	9	4	11	13	20	18	43	30	76	56	103	56	185	17	39
	Tetracyclics																		
N06AA21	Maprotiline	1	5	1	3	0	2	0	0	0	0	0	0	0	0	0	0	0	0
N06AX03	Mianserin	20	51	4	1	1	3	4	5	4	4	6	12	1	17	0	9	0	0
N06AX11	Mirtazapine	296	512	12	2	8	2	15	9	21	13	35	46	55	104	121	231	29	105
	**Total**	**317**	**568**																
	Tricyclics																		
N06AA04	Clomipramine	18	65	1	3	1	1	4	5	2	10	1	19	5	23	4	4	0	0
N06AA06	Trimipramine	3	4	1	1	1	1	1	1	0	1	0	0	0	0	0	0	0	0
N06AA09	Amitriptyline	112	305	1	2	4	9	9	11	7	19	19	32	24	71	42	127	6	34
N06AA10	Nortriptyline	3	8	1	1	1	1	1	1	0	3	0	2	0	0	0	0	0	0
	**Total**	**136**	**382**																
	SNRI																		
N06AX21	Duloxetine	29	66	3	2	4	10	4	4	1	15	4	15	6	18	7	2	0	0
N06AX16	Venlafaxine	69	137	4	1	5	10	6	9	11	16	18	20	12	34	11	38	2	9
	**No CYP2D6 metabolism**																		
N06AG02	Moclobemide	1	0	1	0														
N06AX12	Bupropion	20	19	1	3	1	6	1	5	4	2	8	3	3	0	2	0	0	0
N06AX18	Reboxetine	4	8	1	1	2	2	1	3	0	1	9	1	0	0	0	0	0	0
	**Total**	**25**	**27**																

Supplementary 1 (B). Use of antipsychotic substances stratified for men and women in 10-year age groups.

ATC code	Antipsychotics			20-29 years		30-39 years		40-49 years		50-59 years		60-69 years		70-79 years		80-89 years		90- years	
		Men (total)	Women (total)	Men	Women	Men	Women	Men	Women	Men	Women	Men	Women	Men	Women	Men	Women	Men	Women
	CYP2D6 metabolism with active metabolites																		
N05AX08	Risperidone	159	304	1	1	1	1	4	4	9	13	32	57	81	145	31	83	0	0
N05AX12	Aripiprazole	7	11	1	1	1	1	1	2	1	2	2	4	1	1	0	0	0	0
	**Total**	**166**	**315**																
	Major CYP2D6 metabolism																		
N05AB03	Perphenazine	17	21	3	1	3	1	4	3	5	10	2	5	0	1	0	0	0	0
	Partial CYP2D6 metabolism																		
N05AA01	Chlorpromazine	1	1	1	1	0	0	0	0	0	0	0	0	0	0	0	0	0	0
N05AD01	Haloperidol	106	187	1	1	1	5	4	10	7	42	25	85	59	44	9	0	0	0
N05AF05	Zuclopenthixol	14	28	3	1	1	3	4	3	2	8	4	13	0	0	0	0	0	0
	**Total**	**121**	**216**																
	No CYP2D6 metabolism																		
N05AA02	Levomepromazine	37	45	2	3	1	1	1	5	5	4	5	9	10	7	13	10	0	6
N05AB04	Prochlorperazine	7	24	1	1	1	1	2	4	3	4	0	9	0	5	0	0	0	0
N05AD03	Melperone	11	16	1	5	9	10	1	1	0	0	0	0	0	0	0	0	0	0
N05AE04	Ziprasidone	1	3	1	2	0	1	0	0	0	0	0	0	0	0	0	0	0	0
N05AF01	Flupentixol	12	34	1	2	1	5	2	5	2	11	4	7	2	4	0	0	0	0
N05AF03	Chlorprotixen	6	2	2	2	1	0	2	0	1	0	0	0	0	0	0	0	0	0
N05AH02	Clozapine	16	4	1	1	3	1	1	1	5	1	6	0	0	0	0	0	0	0
N05AH03	Olanzapine	48	73	4	1	7	5	6	2	6	4	8	10	6	21	8	19	3	11
N05AH04	Quetiapiine	29	35	1	1	1	2	2	1	1	3	16	4	7	14	1	8	0	2
N05AX13	Paliperidone	1	0	1	0	0	0	0	0	0	0	0	0	0	0	0	0	0	0
	**Total**	**162**	**232**																
