# Supplementary material for: CYP2D6-inhibiting drugs and risk of fall injuries after newly initiated antidepressant and antipsychotic therapy in a Swedish, register-based case-crossover study
Source: Sci Rep. 2021 Mar 11;11:5796. doi: 10.1038/s41598-021-85022-x (PMC7970948; doi:10.1038/s41598-021-85022-x)
Supplement: Supplementary file 2 — Supplementary Figures. [file 41598_2021_85022_MOESM2_ESM.docx]

**Cover page Scientific Reports**

**CYP2D6-inhibiting drugs and risk of fall injuries after newly initiated antidepressant and antipsychotic therapy in a Swedish, register-based case-crossover study**

Marja-Liisa Dahl^1#^, Karin Leander^2#^, Max Vikström^2^, Clara Frumerie^1^, Sofia Nordenmalm^1^, Jette Möller^3^, Karin Söderberg-Löfdal^1*^

1. Division of Clinical Pharmacology, Department of Laboratory Medicine, Karolinska Institutet, Karolinska University Hospital, Stockholm, Sweden
2. Institute of Environmental Medicine, Karolinska Institutet, Stockholm, Sweden
3. Department of Global Public Health, Karolinska Institutet, Stockholm, Sweden

E-mail address of the corresponding author: [karin.soderberg.lofdal@ki.se](mailto:karin.soderberg.lofdal@ki.se)

#Marja-Liisa Dahl and Karin Leander have contributed equally.

**Supplementary 2.** **Descriptive data of antidepressive (a-c) and antipsychotic (d-f) dispensations in the Swedish population aged 20 years or older in 2006 and 2013 per 10 000 inhabitants categorized for:**

A) the 20 municipalities with the lowest and highest incidence of fall injuries per 10 000 inhabitants

B) the 20 municipalities with the lowest and highest proportion of the population with at least three years of post-secondary education

C) the 20 municipalities with the lowest and highest proportion of foreign-born inhabitants

D) the 20 municipalities with the lowest and highest incidence of fall injuries per 10 000 inhabitants.

E) the 20 municipalities with the lowest and highest proportion of the population with at least three years of post-secondary education

F) the 20 municipalities with the lowest and highest proportion of foreign-born inhabitants

A) the 20 municipalities with the lowest and highest incidence of fall injuries per 10 000 inhabitants (antidepressants)

B) the 20 municipalities with the lowest and highest proportion of the population with at least three years of post-secondary education (antidepressants)

C) the 20 municipalities with the lowest and highest proportion of foreign-born inhabitants (antidepressants)

D) the 20 municipalities with the lowest and highest incidence of fall injuries per 10 000 inhabitants. (antipsychotics)

E) the 20 municipalities with the lowest and highest proportion of the population with at least

three years of post-secondary education (antipsychotics)

F) the 20 municipalities with the lowest and highest proportion of foreign-born inhabitants (antipsychotics)
